# Simplifying the diagnosis of left ventricular hypertrophy: is left ventricular mass to volume ratio constant in children throughout growth?

**DOI:** 10.1186/1532-429X-18-S1-P122

**Published:** 2016-01-27

**Authors:** Simon Lee, Nielsen C James, Shubhika Srivastava, Santosh Uppu

**Affiliations:** 1 Pediatric Cardiology, Icahn School of Medicine at Mount Sinai, New York, NY USA; 2 grid.36425.360000000122169681Pediatric Cardiology, Stony Brook University School of Medicine, Stony Brook, NY USA

## Background

Although it is known that left ventricular hypertrophy (LVH) is a predictor of cardiovascular morbidity and mortality, there is no consensus on how LV mass (LVM) should be indexed, particularly in children. The well published difficulties with correctly indexing LVM hamper the confidence in determining the range of normal LVM in growing children. Left ventricular mass to volume ratio (LVMVR) has potential to avoid some of the inherent problems with indexing. Recent literature in adults has also shown a strong association between increasing LVMVR and increased risk of cardiovascular events. We hypothesized that LVMVR remained relatively stable in healthy children and young adults, and could serve as a simple surrogate to identify LVH.

## Methods

Thirty-one patients without cardiac pathology and normal cardiac MRIs (CMR) were identified and designated as the normal LVMVR group (Group 1). We evaluated LVMVR for differences in gender, age, weight, height, BMI, and BSA, by subdividing Group 1 and comparing the mean LVMVR between the two. The demographic data was normally distributed so the median value in each demographic value was used as a cutoff. To determine if LVMVR could discriminate between patients with abnormal LVM, we identified 30 gender, age, weight, height, and BMI-matched patients with a diagnosis of mild or repaired coarctation as a comparison group (Group 2). Patients with a history of coarctation were chosen because they are known to have LVH and increased regional wall thickness secondary to abnormal ventricular-arterial coupling. We also assessed LVM in all patients using CMR-derived LV mass indexed to BSA with normative values obtained from Kawel-Boehm et al. JCMR 2015.

## Results

**Figure 1:**
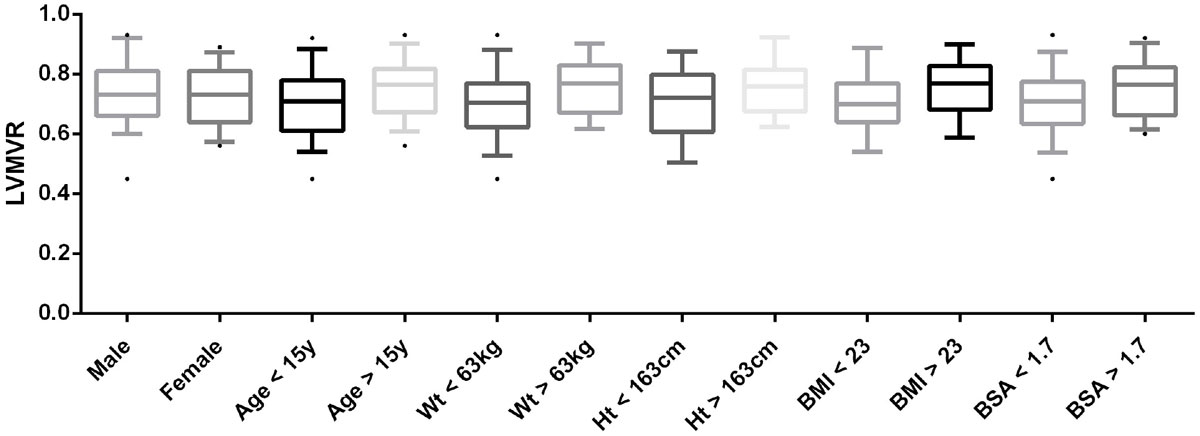
**LVMVR of Group 1 divided by demographics at the 50th percentile**.

Group 1 consisted of 19 males (61%) with a mean age of 15 ± 3.4 years, weight 62.8 ± 19.9 kg, height 163.2 ± 14.4 cm, BMI 23.2 ± 6, and BSA 1.7 ± 0.3. The mean LVMVR of Group 1 was 0.73 g/mL ± 0.11. There were no significant differences in LVMVR by gender (p = 0.88), age (p = 0.55), weight (p = 0.11), height (p = 0.33), BMI (p = 0.15), and BSA (p = 0.22) (Figure 1). No patients had an LVM indexed z-score > +2. Group 2 consisted of 20 males (67%) with a mean LVMVR of 0.88 g/mL ± 0.19 (p = 0.0003). Four patients had LVM indexed z-scores > +2. All 4 had LVMVR Z-scores > +2. Three additional patients were identified with elevated LVMVR z-score who had normal LVM indexed. All patients had a normal LVEDV indexed z-score. No patient in either group had an EF < 55%.

## Conclusions

The CMR-derived LVMVR remains stable in healthy children and young adults and may serve as a more reliable marker of LVH.

